# Correlates of dietary diversity among children aged 6–23 months of head porters in Ghana

**DOI:** 10.3389/fpubh.2022.1020265

**Published:** 2022-11-03

**Authors:** Adwoa Nyantakyiwaa Amoah, Angelina Opoku Danquah, Traore Seydou Stanislav, Emmanuel Kwateng Drokow, Bo Yacong, Ling Wang, Quanjun Lyu

**Affiliations:** ^1^School of Public Health, Zhengzhou University, Zhengzhou, China; ^2^Department of Family and Consumer Sciences, University of Ghana, Accra, Ghana; ^3^Department of Radiation Oncology, Henan Provincial People's Hospital, People's Hospital of Zhengzhou University, Zhengzhou, China; ^4^Faculty of Medicine, Macau University of Science and Technology, Macao, Macao SAR, China

**Keywords:** minimum dietary diversity, knowledge, caregivers, head porters, children

## Abstract

**Objective:**

In many developing countries, most children cannot meet minimum dietary diversity (MDD), defined as the consumption of four or more of the seven food groups. In Ghana, only 35% of children met MDD nationwide in 2017, but rates are worse among the rural poor and resource-constrained individuals like Head Porters (HPs). The current study investigated the correlates of MDD in children of HPs aged 6–23 months old in Ghana.

**Methods and materials:**

A cross-sectional survey was carried out in 2021 among 423 HPs selected purposively from eight market centers in two commercial cities. A multi-stage sampling method was used in obtaining the sample, while a structured interview guide was used to collect data from the caregivers. Stata version 15.1 and descriptive and inferential statistics like frequency, percentage, chi-square and logistic regression were used to analyze the data. All results were deemed significant if the *p*-value was < 0.05 and the odds ratios with a 95% confidence interval.

**Results:**

The children had a mean age of 14.3 (±4.9) months, while half of the caregivers (48.2%) were between 15 and 25 years. Approximately 59% (251) had good knowledge of infant and young child feeding practices (IYCF). About 45% of the children consumed a diversified diet. The number of postnatal care (PNC) visits, delivery in a health facility, meeting minimum meal frequency (MMF), and the child's age was independently associated with MDD at the multivariate level.

**Conclusion:**

Over a third of the caregivers had poor knowledge of IYCF practices. Furthermore, less than half of the children achieved MDD reflecting the need for more education by the stakeholders. Regular PNC visits and delivery in health facilities were independently associated with MDD; therefore, interventions to combat low MDD should prioritize the relevance of these predictors.

## Introduction

According to the 2020 World Health Report, 45.4 million and 149.2 million under-five children were wasted and stunted, respectively. Most of these fatalities are reported in Africa and Southern Asia (1). Again, the World Health Organization (WHO), United Nations International Children's Education Fund (UNICEF), and World Bank estimates of child malnutrition from 2020 showed that over 5 and 30% of African children under 5 years were wasted and stunted (2). The Sustainable Development Goal (SDG) 2 seeks to eliminate all forms of malnutrition and achieve the 2025 global targets on stunting and wasting. Malnutrition has a significant negative impact on the factors that determine a child's quality of life, including physical and intellectual development, school performance, and potential future earnings and productivity (3, 4).

Ghana has made slow but consistent progress in lowering child undernourishment over the past 10 years. The Multiple Indicator Cluster Surveys (MICS) (2017) reported that 20 and 10% of children under 5 years are stunted and underweight, respectively. Again, the latest national dataset conducted in 2014, Ghana Demographic Health Survey (DHS), documented that in 2014, the percentage of stunting, wasting, and under-weight was 19, 5, and 11%, respectively (5). This trend seems promising compared to the malnutrition rates in other African nations. However, these estimations are still deemed high by the WHO (2) and necessitate further research into the root causes of malnutrition in Ghana.

In addition to repeated infections and infectious diseases, inappropriate breastfeeding and suboptimal complementary feeding practices (SCFP) are significant determinants of malnutrition and poor health in Ghana and globally among < 5-year-old children (6–8). According to UNICEF, there is a strong relationship between dietary quality (DQ) and child malnutrition. Dietary diversity (DD) is a valuable indication of children's nutritional status (NS), DQ, and nutrient adequacy (9). It is the number of food types ingested throughout a given time. Minimum dietary diversity (MDD), which measures the percentage of children 6–23 months of age who consumed ≥ 4 food groups the day before, is a critical pointer when dealing with IYCF (10, 11). Nonetheless, meeting the MDD requirement is problematic in many low-income nations because there is evidence from various DHSs undertaken in 33 African countries that only 25% of children could meet the threshold (12, 13). For instance, Burkina Faso and Niger had the worst national MDD rate of 5.6% in 2010 and 8% in 2012, respectively. Also, the MDD prevalence was 19% for Chad in 2015 and DR Congo in 2014, 13% for Ethiopia in 2016, and 22% for Liberia in 2020. Only South Africa had a 50% national prevalence of MDD in the year 2016 (12–14). This phenomenon is attributed to the fact that the diets of most poor households are monotonous since the mainstay is starchy staples like gruel and porridges, which contain few or no animal products, as well as vegetables and legumes (12).

In 2014, ~38% of Ghana's population dwelt in slums or streets, primarily concentrated in commercial cities (15). Among these slums and street-dwellers are uneducated young women who migrate to the cities in the south from the rural north of Ghana due to the high rate of food insecurity, malnutrition, and poverty in the northern regions to work in the informal economic sector as HPs known locally as *kayaye* (15). Therefore, the Ghana Statistical Service (GSS) reported in 2012 that among migrant HPs in Ghana, more than 56% are young school-age girls and women (16).

These girls carry goods on their heads to earn an income due to the lack of convenient transportation in the central business centers between bus terminals and sales points (17). Due to the high sexual activities among these girls, some give birth and stay with their children (17, 18). Children born to such women lack the physical, social and psychological environment needed for proper growth and development and therefore become malnourished (18). Additionally, the dynamic nature of the work stresses the mothers; therefore, breastfeeding rates and child feeding practices are reported to be very low among women in slums, affecting their children's physical growth and development (19). As a result, infant mortality and malnutrition are higher among slum dwellers than in non-slum areas and even rural areas due to the deplorable living conditions of its inhabitants, exposing them to the spread of diseases, poor health, and malnutrition (20).

Studies among HPs previously include Cudjoe and Alhassan, which assessed the social support system of HPs in Kumasi (18), while another study (21) evaluated the utilization of health insurance among female HPs in Kumasi. Also, a study (24) investigated health care inequities affecting female migrants in Ghana, whereas (20) reported cultural and socio-economic difficulties facing female porters in Agbogbloshie, Accra. Clearly, these studies were carried out in small areas and focused mainly on the cultural and socio-economic difficulties (17, 18) and health and living conditions (20). To better understand what is provided and the quality of the food served, we used the UNICEF conceptual framework to assess the multilevel factors that impact the consumption of a diversified diet in children. This study posits that different factors at the individual, maternal, household, environmental and social services relate to enable children achieve MDD (22). Such data will guide policy and help create targeted activities to address the particular issues faced by HPs. To date, no study has been undertaken to ascertain the DD of meals given to UFC of HPs in Ghana. This study, therefore, sought to investigate the correlates of DD among 6–23 months old children of HPs in Ghana.

## Methodology

### Study location

The research was undertaken in Accra and Kumasi, two commercial centers in Ghana, West Africa. According to the 2021 census, Accra is the country's most populated metropolis, with a population of 2.27 million and a land area of 225.67 square kilometers. The city serves as Ghana's economic and administrative center (17, 22). With a population of 1.77 million people and a density of 254 km^2^, Kumasi, Ghana's largest city (after Accra), is a prominent commercial and administrative center, particularly in Ghana's northern and central zones. In Ghana and the sub-region, these two towns are the main business destinations, and their strategic locations also make them a competitive position for HPS. Previous studies (20, 21) documented the following markets Tudu, Agbogbloshei, Mallam Atta, Tema station and, Cocoa Marketing Board in Accra and Adum, Kronum, Race Course, Aboabo, Bantama, and Suame in Kumasi as the markets that HPs operate most in these commercial cities (20).

### Sample size determination

Using the Cochrane formula, the study sample was determined to obtain 500 respondents based on the formula $N=\frac{Z^{2}pq}{e^{2}}$, where N is the desired sample size, Z is the selected critical value of desired confidence level, p is the estimated prevalence of 0.35, q = 1-p, e is the desired level of precision fixed at 5 and 10% estimated non-response rate.

### Sampling procedure

The multistage sampling method was employed. In the first stage, two commercial cities, Accra and Kumasi, were selected to form the primary selection unit. Further, to obtain the secondary selection unit, eight marketplaces documented to have intense head porter activities were chosen using the purposive sampling technique (18). Racecourse, Suame, Bantama, and Kronum were selected from Kumasi, while Agbobloshie, Tudu, Madina, and Tema Station were chosen in Accra. The final stage involved the selection of the various respondents from the various market centers. At this stage, the snowball approach was employed to select eight leaders from each market cluster to determine the population of HPs and a list of those with infants under the age of 2 years in the designated areas, which served as the study's sample frame. The snowball sampling technique is appropriate because previous studies (19, 23, 25) found that HPs lived in groups with a leader and that they have a solid social network, making them reluctant to interact with strangers whom they suspect unless through their leaders, who helped educate and inform them about the study's utility. Furthermore, the fraction method of the systematic sampling technique was used to obtain 63 caregivers each from Agbobloshie, Tudu, Racecourse, and Tema station due to high numbers of HPs present and 62 from the remaining eight market clusters, resulting in a total sample size of 500 caregivers.

### Data collection and statistical analysis

To collect data from the caregivers, a structured interview guide was used. The study tool was translated from the English language into the caregivers' native languages for better understanding. Two investigators independently translated the questions to avoid inconsistencies, and comparisons were made afterward. The guide, adapted from the standard DHS questionnaire, was modified to fit the context of this study. The tool assessed children's, caregivers' obstetrics and health-care characteristics, caregivers' awareness of nutritional diversity and child feeding, and caregivers' DD (26, 27).

After contacting the caregivers through their group leaders, informed consent was given verbally by those who were eligible after informing them of the purpose of the study and assuring them of anonymity. Data was collected on Sundays when the head porters were less busy because of the low economic activities. The data collection process lasted for 6 months, between April and October in 2021. Three trained research assistants facilitated the data collection procedure. Stata 15.1 was used and further entered into a Microsoft Excel database to clean and analyze the data. Means and standard deviations were reported for continuous variables, whiles frequencies with percentages were computed for categorical variables. Pearson's chi-square test of independence was conducted to determine a bivariate statistically significant association between MDD and explanatory variables. Variables found to be statistically significant during the bivariate analysis were included in a multivariate logistic regression model to identify the predictors of the outcome variables. All tests performed were considered statistically significant at *p* < 0.05 at a 95% Confidence Interval.

### MDD determination

Based on the WHO's recommendations, 6–23-month-old children who had four or more foods from the seven standard food groups the day before the study were regarded to have a minimum acceptable DD. The DD score was computed by summing the food categories the child ate the day before the interview. These seven food groups were roots, tubers, and grains; legumes and nuts; all dairy (milk, yogurt, cheese); flesh foods (meat, fish, poultry, and liver/organ meats); eggs; vitamin-A rich fruits and vegetables; other fruits and vegetables (28–30).

The maternal DD was based on the Food and Agriculture Organization's (FAO) Minimum Dietary Diversity for Women (MDD-W), a global pointer of DD among women between 15 and 49-years-old. Ten food groups were summed up to generate the MDD-W score: starchy staples, nuts and seeds, eggs, pulses, flesh foods, dairy foods, leafy green vegetables, other vegetables, other fruits and vegetables rich in vitamin A, and other fruits. Caregivers who consumed foods from ≥ five food groups were considered to have met the MDD-W (31).

### Assessment of nutrition knowledge

Caregivers' knowledge was assessed using a 13-point scale adapted from FAO's Guidelines for assessing nutrition-related Knowledge, Attitudes, and Practices (KAP) manual (32, 33). The categories for knowledge scores were good (≥7) and poor (0–6). The item consisted of questions that solicited the caregiver's nutritional knowledge. A correct response to each question was awarded a score of 1, otherwise 0. The maximum attainable score was 13. A composite knowledge score was calculated for each caregiver, and a median score was determined. Caregivers below the median score (7-points) were considered to have inadequate or poor nutritional knowledge. In contrast, those who scored the median and above (≥7) were classified as having adequate dietary understanding (34).

### Wealth index determination

This was determined using household assets (TV, refrigerator, truck, bicycle, radio, and telephone), housing quality (floor, roofing materials, walls), and water and sanitation facilities. A summation of these gave the proxy WI of each family after creating dummy variables from these and forming terciles. The categorization was poor, middle, and high (34).

## Results

The demographic profile of the study respondents has been presented in Table 1. A total of 423 dyads participated in the study out of the estimated sample of 500, yielding a response rate of approximately 85%. About 48% of the caregivers were between 15 and 25 years of age, while almost 40% had 5–9 members in a household. Nearly 62% of the caregivers had no formal education, and over 83% were married. Muslims constituted about 85% of the total sample. It was revealed that about 50% of the respondents earned 10–20 cedis daily wage, an equivalent of $2.5. Mamprusis formed a predominant part of the respondents comprising almost two-thirds (61.5%) of the total sample.

**Table 1:** Demographic and household characteristics of caregivers and children.

**Characteristics**	**Frequency**	**Percent**
	***N* = 427**	**(%)**
**Demographic characteristics**		
**City of study**		
Accra	213	50.4
Kumasi	210	49.6
Mean ± SD age of caregiver	25.7 ± 5.9	
**Caregivers' age, in years**		
15–24	185	48.1
25–34	160	41.8
35–50	39	10.6
**Marital status**		
Single	44	10.4
Married/living together	363	85.8
Divorced/separated	10	2.4
Widowed	6	1.4
**Highest educational level**		
No education	260	61.5
Primary	138	32.6
Secondary	25	5.9
**Ethnicity**		
Dagomba	58	13.7
Kusasi	12	2.8
Mamprusi	259	61.5
Sesala	36	8.5
Tampauri	8	4.3
Others	40	9.5
**Religion**		
Christianity	57	13.5
Islam	364	86.1
African traditional religion	2	0.4
**No. of years as head porter**		
0–6 mths	147	34.8
7–11 mths	32	7.6
1–2 yrs	60	14.2
>2 yrs	184	43.5
**Daily earning (GHC)**		
< 10	30	7.1
20–30	204	48.2
31–40	142	33.6
>40	47	11.1
**Type of family**		
Single parent	42	9.9
Nuclear	147	34.8
Extended	234	55.7
	**Frequency** **N = 423**	**Percent (%)**
**Household size**		
1–4	105	24.8
5–9	166	39.3
10+	152	35.9
Kiosk	133	31.4
**Wealth index**		
Poorest	117	27.7
Poorer	258	61.2
Richest	47	11.1
**Maternal characteristics**		
**No. of children**		
1–2	213	50.4
3–4	147	34.7
5+	63	14.9
**No. of ANC visits**		
< 4 visits	61	15.3
4+ visits	338	84.7
**Place of delivery**		
Healthcare facility	306	72.3
Others	117	27.7
**Mode of delivery**		
Vaginal delivery	404	95.5
Cesarean section	19	4.5
**No. PNC visits**		
< 4 visits	101	24.7
4+ visits	308	75.3
**Child characteristics**		
Mean child age (in months) ± SD	16.0 ± 7.7 SD	
**Child's age, in months**		
6–11	142	33.6
12–23	281	66.4
**Sex of child**		
Male	209	49.4
Female	214	50.6
**Birth order of child**		
First	100	23.6
Second	135	31.9
Third and above	188	44.5
**Child received BCG immunization**	99	18.1
No	77	18.2
Yes	346	81.8
**Child received Penta 1 and OPV 1**		
No	88	18.2
Yes	346	81.8
**Child received Penta 2 and OPV 2**		
No	101	23.9
Yes	322	76.1
**Child received Penta 3 and OPV 3**		
No	123	29.1
Yes	300	70.9
**Child received measles immunization**		
No	189	44.7
Yes	234	55.3

1 Ghana cedi = ~0.75 USD as at August, 2022.

About 50% had 1–2 children, while 99% were the children's mothers. Over 70% of the children were delivered at a healthcare facility, while about a quarter was born at home. The ante-natal (ANC) and PNC visit rates were 94 and 97%, respectively, among the caregivers. The mean age of the children was 14.3 (±4.9) months. Regarding sex distribution, the females (50.2%) were a little more than the males (49.8%).

### Caregivers' knowledge in IYCF practices

Results of Caregivers' knowledge of IYCFP are presented in Table 2 below.

**Table 2:** Maternal knowledge of IYCF.

**Knowledge variables**	**Frequency (%)**
The first food for a newborn is breast milk only	376 (88.9)
Breastfeeding should be initiated within 1 h after birth	238 (56.3)
A child under 6 months old should be breastfed on demand	383 (90.5)
Expressed Breast milk should be fed to a child in the absence of the mother	20 (4.7)
Cessation of breastfeeding is at 2 years	226 (53.4)
A child should be breastfed on-demand after weaning	296 (70)
A child could be weaned at 6 months of age	249 (58.9)
A child between 6 and 8 months old should be fed at least twice a day	375 (88.7)
A child between 9 and 23 months old should be fed three or more times a day	336 (79.4)
There are 6 Ghanaian food groups a child's meal should be planned with	21 (5.0)
Awareness of signs of Iron-Deficiency Anemia	42 (9.9)
Awareness of causes of Anemia	81 (19.1)
The best way to feed complementary foods is by using a cup/plate and spoon	217 (51.3)
**Overall knowledge of IYCF**	
Poor	172 (40.7)
Good	251 (59.3)

Among the 423 caregivers, 52% (217) got more than seven out of the thirteen questions correctly, having a good knowledge of IYCF. In contrast, 48% (199) had poor knowledge of IYCF practices because they could not respond correctly to more than seven. The caregivers' nutritional knowledge mean score was 5.5 (±1.4) out of 13. Furthermore, 370 (91%) and 357 (87.1%) were knowledgeable about the first food for an infant and the best time to initiate breastfeeding, respectively. As high as 84, 91, and 80% of the respondents lacked knowledge of the existence, signs, and causes of iron deficiency anemia, respectively. Knowledge about the number of food groups a child is supposed to be fed from was the least because < 5% responded correctly. Again, < 5% of the caregivers knew about breastmilk expression.

Concerning sources of information for IYCF, about half (50.4%) and one-third (34.9%) of the respondents mentioned health professionals and relatives, respectively, as the primary source.

### Dietary diversity of 6–23-month-old children

**Figure 1:**
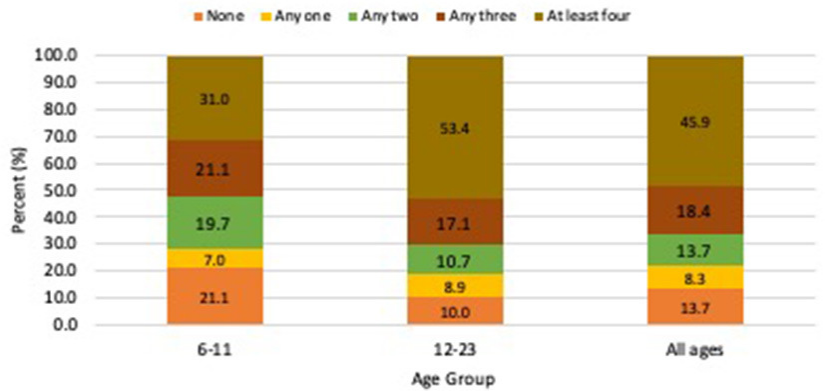
Minimum dietary diversity among children aged 6–23 months.

In Figure 1 and Table 3, minimum dietary diversity among the children and the food groups they consumed the preceding day has been ranked in and ascending order and presented, respectively.

**Table 3:** Food groups consumed the preceding 24-h by children 6–23 months.

**Item**	**Yes (%)**	**No (%)**
Roots, tubers and grains	336 (79.4)	87 (20.6)
All dairy^a^	248 (58.6)	175 (41.4)
Vitamin A-rich fruits and vegetables^b^	242 (57.2)	181 (42.8)
Flesh foods^c^	193 (45.6)	230 (54.4)
Eggs	165 (39.1)	258 (60.9)
Legumes and nuts	121 (28.6)	302 (71.4)
Other fruits and vegetables	95 (22.5)	328 (77.5)

a Includes all dairy, yogurt, cheese, milk and other milk products.

b Includes all pumpkin, carrots, squash or sweet potatoes, dark green leafy vegetables, mangoes, pawpaw, and other locally grown fruits and vegetables that are rich in vitamin A.

c Includes all types of meat, poultry, fish, shellfish, and organ meat.

The measure of MDD was based on a child's ability to consume four or more of the seven food groups as recommended by WHO. The results in Figure 1 revealed that more than half (54.1%) of the children could not meet the MDD since just 45.9% had consumed a diversified diet in the preceding 24-h period. Moreover, the younger the child, the less likely they were to meet the MDD because more infants aged 6–11months (69 %) were unable to meet the MDD compared to those between 12 and 24 months old (46.6%), as presented in Figure 1. Among the seven food groups, fruits and vegetables (22.5%) were the least consumed fruits and vegetables. Equally, as can be observed in Table 3, the intake of legumes and eggs was low because this was lacking in the meals of over 70 and 60% of the children, respectively. Grains and tubers were the highest consumed food group (81%), followed by milk and dairy foods (61.5%). Moreover, < 40% reported consuming eggs in the past 24 h.

### Association between demographic and household characteristics of caregivers and children and MDD

The association between demographic and household characteristics of the caregivers and their children and MDD has been presented in Table 4.

**Table 4:** Association between maternal and child characteristics and MDD among children 6–23 months.

**Items**		**MDD**			
	**No**	**Yes**	**No**	**Chi-**	**p-**
	**(%)**	**(%)**	**(%)**	**square**	**value**
**Demographic characteristics**					
**City**					
Accra	213 (50.4)	103 (50.1)	110 (48.0)	1.070	0.300
Kumasi	210 (49.6)	91 (46.9)	119 (52.0)		
**Caregivers' age, in years**					
15–24	185(48.2)	83 (46.6)	102 (49.5)	0.56	0.754
25–34	160 (41.7)	75 (42.1)	85(41.3)		
35–50	39 (10.6)	20 (11.2)	19 (9.2)		
**Marital status**				3.46	0.326
Single	44 (10.4)	16 (8.3)	28(12.2)		
Married/living together	363 (85.8)	173(89.8)	190 (83)		
Divorced/separated	10 (2.4)	3 (1.6)	7 (3.1)		
Widowed	6 (1.4)	2 (1.0)	4 (1.8)		
**Highest educational level**				1.87	0.393
No Education	260 (61.5)	126 (65.0)	134 (58.5)		
Primary	138 (32.6)	58 (29.9)	80 (34.9		
Secondary	25 (5.9)	10 (5.1)	15 (6.6)		
**Ethnicity**					
Dagomba	58 (13.7)	20 (10.3)	38 (16.6)	23.08	**0.0003**
Kusasi	12 (2.8)	5 (2.6)	7 (3.1)		
Mamprusi	259 (61.2)	139 (71.7)	120 (52.4)		
Sesala	36 (8.5)	6 (3.1)	30 (13.1)		
Tampauri	18 (4.3)	9(4.64)	9 (4.64)		
Others	40 (9.5)	15 (7.7)	25 (10.9)		
**Religion**					
Christianity	57 (13.48)	26 (13.4)	31 (13.5)	1.71	0.426
Islam	364 (86.1)	168 (86.6)	196 (85.6)		
African traditional religion	2 (0.47)	0 (0.00)	2 (0.9)		
**No. of years as head porter**				12.34	**0.00063**
0–6 mths	147 (34.8)	58 (29.9)	89 (38.7)		
7–11 mths	32 (7.6)	8 (4.12)	24 (10.5)		
1–2 yrs	60 (14.2)	32 (16.5)	28 (12.2)		
>2 yrs	184 (43.5)	88 (38.4)	96 (49.5)		
**Average amount of money made daily**					
< 10	30 (7.1)	11 (5.7)	19 (8.3)	2.26	0.521
10–20	204 (48.2)	92 (47.4)	112 (48.9)		
30–40	142 (33.6)	71 (36.6)	71 (31.6)		
>40	47 (11.1)	20 (10.3)	27 (11.8)		
**Type of family**				4.62	0.099
Single Parent	42 (9.9)	13 (6.7)	29 (12.7)		
Nuclear	147 (34.8)	73 (37.6)	74 (32.3)		
Extended	234 (55.3)	108 (55.7)	126 (55.0)		
**Household size**				1.03	0.598
1–4	105 (24.8)	44 (22.7)	61 (26.6)		
5–9	166 (39.2)	80 (41.2)	86 (37.6)		
10+	152 (35.9)	70 (36.1)	82 (35.8)		
**Wealth index**				2.09	0.352
Poorest	117 (27.7)	59 (30.4)	58 (25.4)		
Poorer	258 (61.1)	117 (60.3)	141 (61.8)		
Richest	47 (11.4)	18 (9.3)	29 (12.7)		
**Maternal characteristics**					
**Knowledge of IYCF**				**0.86**	**0.031**
Good	251 (56.3)	116 (59.8)	135 (58.9)		
Poor	172 (40.7)	78 (40.2)	94 (41.1)		
**No. children**				1.47	0.4805
1–2	213 (50.35)	92 (47.4)	121 (52.8)		
3–4	147 (34.8)	73 (37.6)	74 (32.3)		
5+	63 (14.9)	29 (15.0)	34 (14)		
Aunt	3 (0.71)	1 (0.52)	2 (0.87)		
**Met maternal dietary diversity**				5.477	0.019^*^
No	174 (41.1)	68 (35.1)	106 (46.3)		
Yes	249 (58.9)	126 (64.9)	123 (53.7)		
**No. ANC visits**				2.05	0.1522
< 4 visits	61 (15.3)	23 (12.5)	38 (17.7)		
4+ visits	338 (84.7)	161 (87.5)	177 (82.3)		
**Place of delivery**				8.47	0.0036^*^
Healthcare facility	306 (72.3)	127 (65.5)	179 (78.2)		
Others	117 (27.66)	67 (34.5)	50 (21.8)		
**Mode of delivery**					
Vaginal delivery	404(95.5)	187 (96.4)	217 (94.8)	0.65	0.419
Cesarean section	19 (4.5)	7 (3.61)	12 (5.24)		
**No. PNC Visits**					
< 4 visits	101(24.7)	36 (19.2)	65 (29.4)	5.75	0.0165^*^
4+ visits	308(75.3)	152 (80.9)	156 (70.6)		
**Child characteristics**					
**Child met MMF**					
No	229 (54.1)	111 (39.5)	72.21	0.000^*^	
Yes	194 (45.9)	170 (60.5)			
**Child's age, in months**					
6–11	142 (33.6)	44 (22.7)	19.05	0.000^*^	
12–23	281 (66.4)	150 (77.3)			
**Sex of child**					
Male	209 (49.4)	94 (48.5)	0.13	0.718	
Female	214 (50.6)	100 (51.6)			
**Birth order**					
First	100 (23.6)	46 (23.7)	0.04	0.980	
Second	135 (31.9)	61 (31.4)			
Third and above	188 (44.4)	87 (44.9)			
**Child received BCG immunization**					
No	77 (18.2)	24 (12.4)	8.19	0.0042^*^	
Yes	346 (81.8)	170 (87.6)			
**Child received Penta 1 and OPV 1**					
No	88 (20.8)	28 (14.4)	8.83	0.003^*^	
Yes	335 (79.2)	166 (85.6)			
**Child received Penta 2 and OPV 2**					
No	101 (23.9)	36 (18.6)	5.58	0.0182^*^	
Yes	322 (76.1)	158 (81.4)			
**Child received Penta 3 and OPV 3**					
No	123 (29.1)	42 (21.7)			
Yes	300 (70.9)	152 (78.4)	9.59	0.0020^*^	
**Child received measles**					
No	189 (44.7)	75 (38.7)			
Yes	234 (55.3)	119 (61.3)	5.26	0.0219^*^	

* *p*-value less than 0.05. Bold values means *p*-values which are statistically significant.

A Pearson's chi-square of independence test was performed at a statistically significant threshold of α = 0.05 to determine factors associated with MDD (Table 4). The test found a statistically significant difference between caregivers' ethnicity, the number of years as a head porter (HP), maternal DD, place of baby delivery, PNC visits, child meeting MMF, child's age, BCG, Penta1, Penta 2, Penta 3, and measles immunization.

### Determinants of MDD among children 6–23 months

The independent variables that were statistically significant in the bivariate analysis were modeled into the multivariate regression to estimate the determinants of MDD among children aged 6–23 months. The findings are reported in Table 5.

**Table 5:** Determinants of MDD among children 6– 23 months.

**MDD**	**Adjusted odds ratio**	**95% CI**	**p-value**
**Ethnicity**			
Dagomba	Reference		
Kusasi	1.20	[0.28–5.03]	0.807
Mamprusi	1.36	[0.71–2.61]	0.358
Sesala	0.41	[0.14–1.22]	0.108
Tampauri	1.45	[0.46–4.64]	0.526
Others	0.768	[0.31–1.91]	0.572
**No. years as a head porter**			
0–6 mths	Reference		
7–11 mths	0.36	[0.15–0.96]	**0.050**
1–2 yrs	1.27	[0.65–2.49]	0.483
>2 yrs	1.03	[0.63–1.72]	0.881
**Place of child delivery**			
Others	Reference		
Healthcare facility	2.03	[1.25–3.31]	**0.004**
**No. PNC visits**			
< 4 visits	Reference		
4+ visits	1.82	[1.09–3.05]	**0.023**
**Age of child (months)**			
6–11	Reference		
12–23	2.39	[1.48–3.87]	**0.000**
**Maternal dietary diversity**			
No	Reference		
Yes	1.45	[0.92–2.28]	0.102
**Knowledge of IYCF**			
Poor	Reference		
Good	1.02	(0.66–1.58)	0.08
**Child received BCG immunization**			
No	Reference		
Yes	1.22	[0.35–4.31]	0.756
**Child received Penta 1 and OPV 1 immunization**			
No	Reference		
Yes	1.18	[0.32–4.37]	0.800
**Child received Penta 2 and OPV 2 immunization**			
No	Reference		
Yes	0.40	[0.11–1.44]	0.162
**Child received Penta 3 and OPV 3 immunization**			
No	Reference		
Yes	0.93	[0.41–2.14]	0.868
**Child received measles immunization**			
No	Reference		
Yes	1.15	[0.67–1.96]	0.617

Bold values means *p*-values which are statistically significant.

The number of years as a head porter (HP), the place of baby delivery, the number of PNC visits, meeting MMF, and the child's age all had a statistically significant relationship with MDD. Compared to individuals who had been HPs for < 7 months, those who were HPs for 7–11 months were 64% (AOR = 0.36; 95% CI: 0.15; 0.96, *p* = 0.046) less likely to meet the MDD. Compared to caregivers who delivered elsewhere, those who delivered in a health institution were more than twice as likely to meet the MDD. Again, caregivers who made more than four (≥4 visits) PNC visits were 1.8 (AOR = 1.82; 95% CI 1.09–3.05; *p* = 0.023) times more likely to feed the MDD than caregivers who made fewer than four PNC visits. Moreover, children between 12 and 23 months and those who met the MMF had 2.4 (AOR = 1.82; 95% CI 1.09–3.05; *p* = 0.023) and 2.3 (AOR = 1.82; 95% CI 1.09–3.05; *p* = 0.023) higher odds of meeting the MDD than their younger counterparts of 6–11 months and who did not meet the MMF, respectively. However, all the remaining variables were not statistically significant at the multivariate level.

## Discussions

The analysis showed that a little over half of the caregivers were between 15 and 25 years which reflects the young age of the caregivers and confirms previous studies among HPs (35, 36), in which approximately 80% of the respondents were between 15 and 24 years. Additionally, earlier studies among HPs indicated that over 80% were uneducated, a trend similar to our findings. This could be attributed to the belief that in some socio-cultural settings, females are socialized into homemaker roles. Therefore, education may not be a priority for them (36). The wages earned by the caregivers were significantly inadequate. This could affect their access to nutritious food and compromise quality food consumption and adequate quantities needed for the physiological functions of their children (36).

The knowledge level of the caregivers was relatively low, given that not even one percent obtained more than 10 out of the 13 questions posed to them. Probably, the low educational level explains this outcome because the knowledge, either subjective or objective, is a driver that influences food choice and impacts the adoption of healthy eating behavior. Again, nutrition knowledge is associated with a quality diet and could enable caregivers to obtain accurate information on what should be fed to the child and its effects on health, thereby improving food diversity. Our results confirm a study (33) conducted in a similar socioeconomic setting in Ghana. Of great concern is the poor knowledge of the caregivers on the number of food groups children should be fed from. Since over 90% did not know these food groups, one would wonder how proper planning of the child's meal will be done to achieve a diversified meal. This result suggests that the Ghana Health Service (GHS) and other stakeholders should intensify nutrition education at ANC and PNC. Moreover, knowledge of the causes and signs of Iron Deficiency Anemia (IDA) was shallow. In Ghana, over 70% of under-5-year-olds are anemic (4); hence the provision of knowledge at health facilities, radios, and religious facilities may improve the knowledge base of these caregivers.

The results revealed that the prevalence of MDD was 45.9%, and this rate is slightly lower than the 47% rate reported in the Ghana Multiple Indicator Cluster Survey (MICS4) (37) but higher than the GDHS rate of 27% (38). Differences in study design, timing, and environment could account for the disparity. Moreover, it was discovered that MDD increased with age, which is comparable to earlier studies (36, 38) in Ghana and elsewhere in Ethiopia (30) and Uganda (42). This finding implies that these younger children may be unable to meet their nutrient needs. Therefore, more attention should be given to the younger children who are beginning the weaning process by caregivers since they are more vulnerable.

The food group, fruits and vegetables, was least consumed. This could be because of the cost, which may be the high cost which is unaffordable for individuals from poor households in Ghana, or caregivers' lack of knowledge on how to incorporate them into meals and hence the need for education. Studies in Ethiopia (30, 39), Ghana (40, 41), and a comparative study in Vietnam, Bangladesh, and Ethiopia (43) all found a similar pattern. The low intake of legumes and eggs is problematic, considering the unique roles they play in the development and growth of a child. The low consumption could be attributed to some myths and beliefs associated with the intake of protein foods like eggs among some ethnic groups, as reported in studies undertaken in Ghana, Nigeria, and Ethiopia (44–47). Importantly, these are relatively cheaper protein sources for a disadvantaged group like the HPs. Our results call for more education by stakeholders on the usefulness of affordable but nutritious foods among low-income groups during ANC and PNC visits.

The number of PNC visits, an underlying determinant of the model used for this study was a predictor of MDD. This finding is consistent with previous studies (30, 44, 46). All these studies revealed that caregivers who attended PNC visits were much more likely to achieve MDD than their counterparts who did not. The positive association could mean that these caregivers practice education obtained during PNC visits.

Baby delivery in a health facility was positively associated with providing a diversified diet. This result corroborates with studies in Ethiopia and South Asia (48, 49) and could be explained by the fact that caregivers are well educated during ANC visits by health personnel on the need to feed a diversified diet. Children that met the MMF were far more likely to meet the MDD, which aligns with previous reports (30, 50). This is expected since meeting MMF meant eating the recommended number of times, which could include a more diversified diet.

A limitation of this analysis is the cross-sectional nature, so the data cannot establish causal relationships. Also, self-reports of the caregivers could be a source of recall bias. The children's dietary intakes were determined with a 24-h recall, but this may not be a true reflection of their nutritional habits and intakes.

## Conclusion

More than a third of the caregivers had poor knowledge of IYCF practices. Furthermore, less than half of the children achieved MDD reflecting the need for more education by the stakeholders. The study confirmed regular PNC visits and delivery in health facilities as independently associated with MDD; therefore, interventions to combat low MDD should prioritize the relevance of these predictors in order to alleviate morbidity, mortality, and under-nutrition in this population which can enable Ghana to achieve its national nutritional targets. Stakeholders like the GHS should develop innovative ways to reach out to these caregivers given their busy daily schedules. This could include organizing ANC and PNC roadshows for the mothers on Sundays and holidays in order to encourage them to participate.

## Data availability statement

The raw data supporting the conclusions of this article will be made available by the authors, without undue reservation.

## Ethics statement

The studies involving human participants were reviewed and approved by the Institutional Review Board (IRB) of the School of Public Health, Zhengzhou University (ZZUIRB 2020-58). Written informed consent to participate in this study was provided by the participants' legal guardian/next of kin.

## Author contributions

AA and QL developed the study framework, performed data analysis, and drafted the paper. The data analysis inputs were provided by AD and LW with support from TS, ED, and BY. All authors reviewed and contributed to subsequent drafts and approved the final version for publication.

## Conflict of interest

The authors declare that the research was conducted in the absence of any commercial or financial relationships that could be construed as a potential conflict of interest.

## Publisher's note

All claims expressed in this article are solely those of the authors and do not necessarily represent those of their affiliated organizations, or those of the publisher, the editors and the reviewers. Any product that may be evaluated in this article, or claim that may be made by its manufacturer, is not guaranteed or endorsed by the publisher.
